# The Determination of Sex

**Published:** 1876-09

**Authors:** 


The Determination of Sex.—Prof. Mayerhofer, of Vienna, considers that the man and woman are equally concerned in influencing the sex, each having a tendency to reproduce its own sex. If the male is young and strong, and not given to venereal excess, the probability is greater that a male child will be born; in the opposite case a female. Domestic animals (the cow), fertilized at the commencement of "heat," bear more females than males; the opposite is the case at the end of "heat."—*Med. and Surg. Reporter.*

